# Mitochondrial Modulations, Autophagy Pathways Shifts in Viral Infections: Consequences of COVID-19

**DOI:** 10.3390/ijms22158180

**Published:** 2021-07-30

**Authors:** Shailendra Pratap Singh, Salomon Amar, Pinky Gehlot, Sanjib K. Patra, Navjot Kanwar, Abhinav Kanwal

**Affiliations:** 1 Department of Pharmacology, New York Medical College, Valhalla, NY 10595, USA; 2 Department of Microbiology and Immunology, New York Medical College, Valhalla, NY 10595, USA; 3 Department of Pharmacy, School of Chemical Sciences and Pharmacy, Central University of Rajasthan, Bandarsindri, Kishangarh, Ajmer 305817, Rajasthan, India; gehlotpinky07@gmail.com; 4 Department of Yoga, Central University of Rajasthan, Bandarsindri, Kishangarh, Ajmer 305817, Rajasthan, India; sanjib.patra@curaj.ac.in; 5 Departmen of Pharmaceutical Sciences and Technology, Maharaja Ranjit Singh Punjab Technical University, Bathinda 151001, Punjab, India; kanwar_navjot@yahoo.com; 6 Department of Pharmacology, All India Institute of Medical Sciences, Bathinda 151001, Punjab, India; abhinavkanwal@gmail.com

**Keywords:** mitochondria, SARS-COV2, potential targets, autophagy, COVID-19, viral infections

## Abstract

Mitochondria are vital intracellular organelles that play an important role in regulating various intracellular events such as metabolism, bioenergetics, cell death (apoptosis), and innate immune signaling. Mitochondrial fission, fusion, and membrane potential play a central role in maintaining mitochondrial dynamics and the overall shape of mitochondria. Viruses change the dynamics of the mitochondria by altering the mitochondrial processes/functions, such as autophagy, mitophagy, and enzymes involved in metabolism. In addition, viruses decrease the supply of energy to the mitochondria in the form of ATP, causing viruses to create cellular stress by generating ROS in mitochondria to instigate viral proliferation, a process which causes both intra- and extra-mitochondrial damage. SARS-COV2 propagates through altering or changing various pathways, such as autophagy, UPR stress, MPTP and NLRP3 inflammasome. Thus, these pathways act as potential targets for viruses to facilitate their proliferation. Autophagy plays an essential role in SARS-COV2-mediated COVID-19 and modulates autophagy by using various drugs that act on potential targets of the virus to inhibit and treat viral infection. Modulated autophagy inhibits coronavirus replication; thus, it becomes a promising target for anti-coronaviral therapy. This review gives immense knowledge about the infections, mitochondrial modulations, and therapeutic targets of viruses.

## 1. Introduction

Mitochondria are membrane-bound cell organelles which produce energy in the form of adenosine triphosphate (ATP). Mitochondria regulate various intracellular functions like metabolism, bioenergetics, cell death, innate immune signaling, and cellular homeostasis [1].

Mitochondrial dynamics and mitochondria, selective autophagy, or mitophagy, work to maintain mitochondrial quality control [2]. By altering mitochondrial dynamics, viruses influence innate immune signaling [which is mediated through the mitochondrial antiviral signaling (MAVS) protein], as well as favoring their propagation by taking advantage of mitochondrial metabolite.

### 1.1. Mitochondrial Dynamics

The mitochondrial dynamics network involves two cycles, mitochondrial fission and Mitochondrial Fusion, to help maintain the functional capacity of mitochondria by distribution of mitochondrial contents, energy conductance, and responsiveness to cellular cues. Thus, mitochondrial dynamics govern their communication and interaction with other cellular organelles.

#### 1.1.1. Mitochondrial Fission

Mitochondrial fission is required to create new mitochondria, segregate damaged parts of the mitochondria from the dynamic mitochondrial network and remove damaged mitochondria via the mitochondria-selective autophagy process. Dynamin-1 like protein (Drp1) recruitment into mitochondria and its activity is regulated by different processes such as phosphorylation, nitrosylation, and summoylation to initiate mitochondrial fission. In mammals at least three proteins are required for mitochondrial fission: dynamin-related protein 1 (Drp1), Fis1 mitochondrial fission 1 protein [Fis1), and mitochondrial fission factor (MFF) [3,4]. Drp1 contains three domains: the dynamic like central domain, C-terminal GTPase effector domain, and N-terminal GTPase domain. Full GTPase efficiency and mitochondrial fission requires intermolecular interaction between the GTPase domain and GTPase effector domain [5]. By network lengthening, MFF releases the Drp1 foci from the mitochondrial outer membrane, whereas, with the help of mitochondrial fission and the physical interaction between MFF and Drp1, MFF overexpression stimulates mitochondrial fission [6].

#### 1.1.2. Mitochondrial Fusion

Mitochondrial Fusion mechanisms involve various steps such as outer mitochondrial membrane (OMM) fusion, and inner mitochondrial membrane (IMM) fusion through integral membrane GTPase proteins such as Mitofusin 1 and 2 (Mfn1 and Mfn2), and optic atrophy 1 (OPA1), respectively. The proteins Mfn1 and Mfn2 are located on the opposite fusion membranes and anchored into the outer membrane with the N-terminal GTPase domain and a predicted coiled coil protruding into cytosol to form homo or hetro-oligomeric complex in trans. The OPA1 protein is located on adjacent fusion membrane and is involved in inner-mitochondrial membrane fusion as well as mitochondria cristae remodeling, apoptosis, and bioenergetics. The OPA1 protein works with Mfn1 to promote mitochondrial fusion. Mitochondrial fusion isolates dysfunctional and damaged mitochondria from the functional network via the joining of healthy discreate mitochondria with the functional network.

#### 1.1.3. Role of Mitochondrial Dynamics in Antiviral Signaling

By balancing between two opposite processes, mitochondrial fission and fusion, mammalian cells maintain the overall shapes of their mitochondria. The Fis1 protein has a TM domain with the help of the C-terminal of mitochondria anchored into the mitochondrial outer membrane [3]. Drp1 does not prevent localized mitochondria via the knockdown of Fis1 with RNA interference [7]. By network lengthening, MFF release the Drp1 foci from the mitochondrial outer membrane, whereas, with the help of mitochondrial fission and the physical interaction between the mitochondrial fission factor (MFF) and Drp1, MFF overexpression stimulates mitochondrial fission [6].

The proteins Mfn1, Mfn2, and OPA are involved in mitochondrial dynamics maintenance, [8,9]. For mitochondrial fusion, OPA1 needs Mfn1 [10] and forms an oligomer that regulates mitochondrial cristae morphology and therefore completely unharnesses the cytochrome C oxidase throughout the process of cell death [9,11,12]. The process of RLR communication reserves the interaction between Mfn2 and MAVS in high-molecular mass complexes [13]. Once a virus infects the mitochondria, Mfn2 murine embryonic cells (MEFs) improve MAVS communication, whereas overexpression of Mfn2 blocks NF-kB and the IRF-3 activation downstream of RIG-I, MDA-5, and MAVS [14]. After the manipulation of its expression level, Mfn1 produces different phenotypes, which indicate that Mfn2 has a unique role in regulating MAVS signaling, which is independent of its function in mitochondrial fusion.

Efficient RLR signaling requires the interaction of MAVS with Mfn1, whereas MFF1 or DRP1, as an inhibitor of fusion, decreases virus-induced NF-kB and IRF-3 activation [15]. After the depletion of Drp1 and Fis1 in the cells, there is an increase in RLR signaling, and the elongation of the mitochondrial network promotes mitochondrial–endoplasmic reticulum interaction during the viral infection, enhancing the association of MAVS with a sting to augment RLR signaling [15].

MAM is a major site of MAVS signaling which links the endoplasmic reticulum to the mitochondria [16], where Mfn2 may inhibit MAVS [17]. After the activation of RLR, both IRF-3 and IKB- α phosphorylate, which degrades the main 75K Da isoform of MAVS resulting in the release of Mfn1 to promote mitochondrial fusion/elongation [15]. Thus, MAVS acts as a regulator of the Mfn1 function. After mitofusion-deficient MFFs [both Mfn1 and Mfn2 proteins), heterogenous mitochondrial membrane potential (MMP) occurs, which reduces MAVS signaling, resulting in a decrease in RLR-dependent antiviral responses.

When cells treated with a chemical uncoupling compound, mitochondrial membrane potential decreases RLR signaling to NF-KB and IRF-3 as well as lowering the production of type 1 IFN [18]. Upon viral infection, decreased MMP might quickly thwart the MAVS complex’s structural rearrangement [18]. Inhibition of ATP synthesis does not inhibit MAVS-mediated signaling, excluding the hypothesis that MAVS, localized at the mitochondrial surface, is not attributed to an energetic requirement to transduce the signal. Mfn1 and Mfn2 have opposite roles in innate viral immunity, whereas they play a similar role in mitochondrial fusion [19].

In the absence of infection, the innate immune system is physiologically activated and produces inflammation, also known as chronic low-grade inflammation [20], which includes genetic susceptibility, cellular senescence, impaired autophagy, dysfunctional mitochondria, changes in microbiota composition, and oxidative stress [21,22,23].

In healthy mitochondria, about 90% of energy demand is provided by mitochondria through ATP generation [24]. The imbalance between ATP supply and demand causes mitochondrial dysfunction.

Mitochondrial fission includes Drp1 and Fis1, whereas mitochondrial fusion includes Mfn1, Mfn2, and OPA1. The deletion of the Drp1 gene causes mitochondrial enlargement, the increased opening of the mitochondrial permeability transition pore (MPTP), apoptosis, and lethal dilated cardiomyopathy (DCM) [25] by inhibiting mitochondrial fission, whereas deletion of Mfn1 and Mfn2 disrupts mitochondrial structure and respiratory chain function [26]. An imbalance between mitochondrial fusion and fission compromises mitochondrial integrity during aging [27,28,29]. Mitochondrial from aged C. elegans is indicated by a significantly enlarged and swollen ultrastructure, which is accompanied by decreasing O2 consumption, increasing carbonylated proteins and decreasing mitochondrial SOD activity [30].

Drp1 knockdown triggers NLRP-3 inflammasome assembly and activates caspase1 & IL-β [31].

The depolarization of membrane and mitochondrial damage is caused by PINK1 (PTEN-induced kinase 1), which accumulates on the outer membrane of the mitochondria, mediates phosphorylation, and activates parkin (E3 ubiquitin ligase) for the ubiquitination of the mitochondrial protein of Mfn-2 [32], resulting in damaged mitochondria interacting with an LC3-positive phagosome for degradation in the lysosome. Thus, if this process is impaired, it leads to mitochondrial dysfunction and cell death [33,34]. The deficiency of parkin increases MMP loss, ROS production, and mtDNA release, which triggers NLRP-3 and elevates the activation of IL-1B and caspase, contributing to age-related pathologies [35,36]. Upregulation of parkin expression and enhanced mitophagy inhibits NLRP-3 inflammasome assembly and activates downstream signaling molecules that promote cell survival [37].

### 1.2. Autophagy

**Figure 1:**
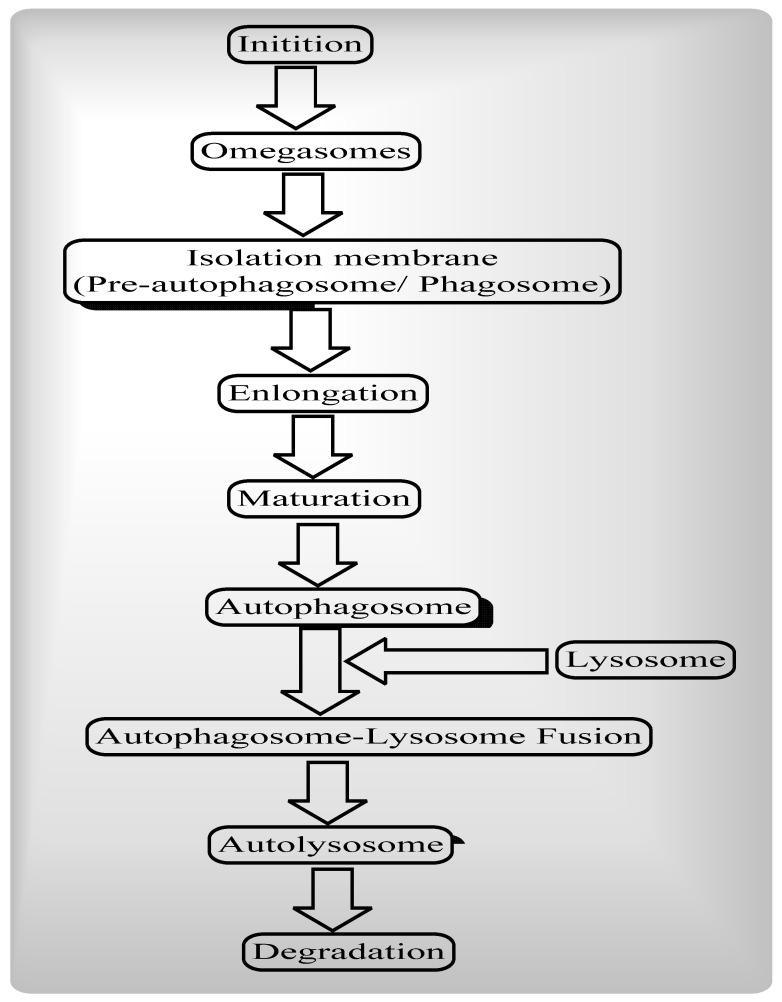
**—Steps of Autophagy:** Autophagy (macro autophagy) means “self” (“auto”) and “eating” (“phagy”). Autophagy removes dysfunctional and unnecessary cellular components through various steps such as Omegasomes and the initiation of isolation membrane; the elongation of the isolation membrane and formation of autophagosomes; and autophagosome-lysosome fusion and degradation.

Autophagy is a self-destructive process which involves the removal of dysfunctional and unnecessary cellular components through various steps such as Omegasomes and the initiation of isolation membrane; elongation of isolation membrane and formation of autophagosomes; and autophagosome-lysosome fusion and degradation (Figure 1).

#### 1.2.1. Omegasomes and the Initiation of Isolation Membrane:

Through the mTOR-signaling pathway, the ULK1-Atg13-FIP200-Atg101 kinase complex activates autophagic signaling and forms omegasomes. Under the starvation condition, double FYVE domain-containing protien1(DFCP1) is localized to PI [3] P on an omegasome whereas, under nutrient-rich conditions, DFCP1 is localized to ER and Golgi. The formation of DFCP1-positive omegasomes is regulated by the Atg14-VPs34-beclin1 PI3-kinase complex. Inside the ring of the omegasome, an isolation membrane is formed, and the Atg12-Atg5/Atg16 complex is localized to this isolation membrane [38,39,40]. By decreasing the level of PI [3] P [41,42], 2 PI [3] P, phosphates such as jumpy/MT/MR14 and MTMR3 negatively regulate the formation of omegasomes and the isolation membrane.

#### 1.2.2. Elongation of the Isolation Membrane and the Formation of Autophagosomes

The isolation membrane engulfs cytoplasmic components and elongates. At the later stage of the elongation of the isolation membrane, LC3-11 is localized to both sides of the isolation membrane. It closes the membrane to form an autophagosome resulting in the Atg12-Atg5/Atg16 complex, dissociated from the autophagosome [40]. Moreover, LC3-II, Rab32, and Rab33 also involve the elongation of the isolation membrane [43,44].

#### 1.2.3. Autophagosome-Lysosome Fusion and Degradation

The outer membrane of the autophagosome fuses with the lysosome to form autolysosome, which requires Rab7 [45,46]. This fusion is positively regulated by the UVRAG-VPS34-Beclin1 PI3-Kinase complex while negatively regulated by the Rubicone-UVRAG-VPS34-Beclin1 PI3-Kinase complex [47,48,49,50,51]. Lysosome hydrolase in autolysosome involves cathepsin, and lipases degrade the intra-autophagosomal content, while cathepsin alone degrades LC3-II on the intra-autophagosomal surface [52,53].

Coronaviruses such as SARS-Co, SARS-CoV-2, MERS-CoV, and MHV, etc., induce as well as inhibit autophagy. Modulated autophagy inhibits coronavirus replication; thus, it becomes a promising target for anticoronaviral treatments (Table 1).

Autophagy inducers antagonize coronavirus replication, whereas autophagy inhibitors disorganize the Golgi and prevent amohisome/autophagosome-lysosome fusion resulting in blocking the vesicle trafficking system [59,75,76].

**Figure 2:**
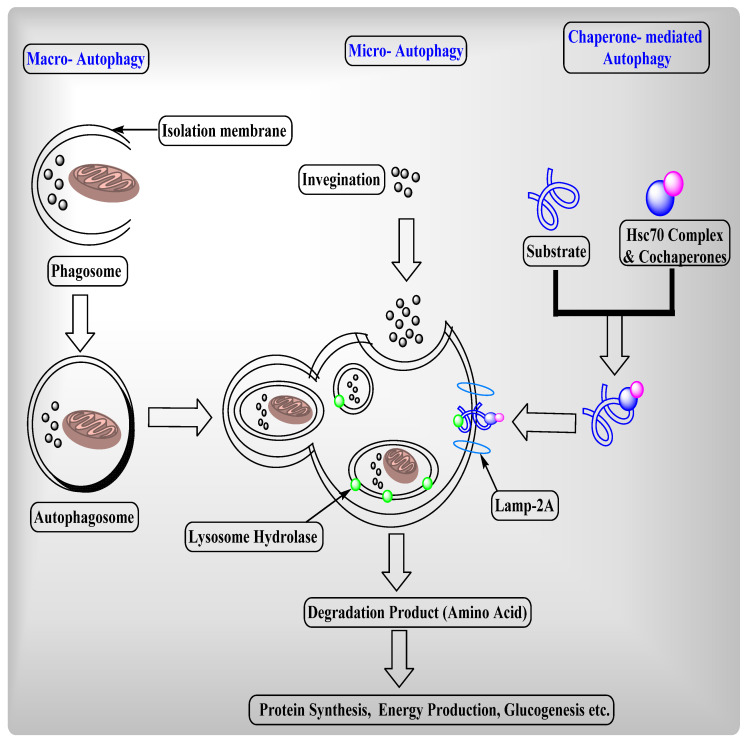
**—Types of Autophagy:** Autophagy categorized into three different categories based on differences in their routes to the lysosome.

In the mammalian cell, autophagy is categorized into three types: macro-autophagy, micro-autophagy, and chaperone-mediated autophagy (Figure 2), on the basis that these types differ in their routes to the lysosome [77]. Both macro-autophagy and micro-autophagy are non-selective degradations of protein, lipid, and organelles, whereas chaperone-mediated autophagy is a selective protein degradation. Chaperone-mediated autophagy has a specific-signal sequence called KFERQ, which depends on the molecular chaperone (Heat schock coganate 70) Hsc70.

**Figure 3:**
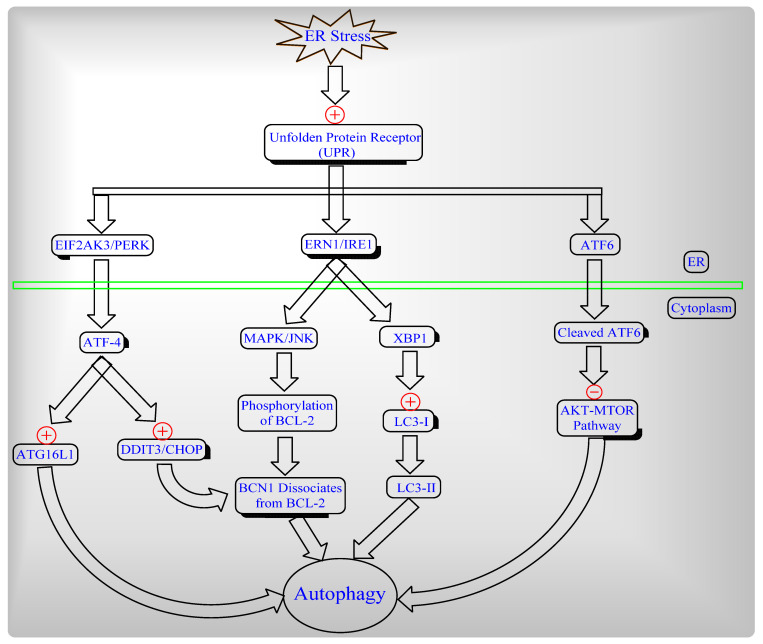
ER stress-induced unfolded protein receptor [UPR] pathways to cause Autophagy.

Autophagy induced by three different levels of ER stress and the unfolded protein receptor (UPR) which branches to EIF2AK3/PERK, ERN/IRE1 and/or the ATF6 signaling pathway (Figure 3).

**Figure 4:**
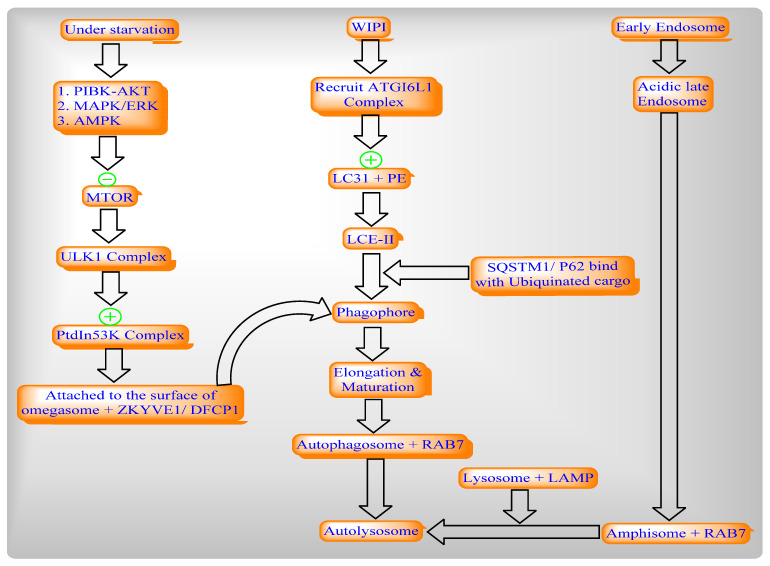
Autophagy pathway including the convergence of the endocytic pathways.

Autophagy conserved the eukaryotic process of cytoplasmic degradation which was activated under different conditions of starvation and endoplasmic reticulum [ER] stress to maintain cellular homeostasis as well as to achieve the complete autophagic flux (Figure 4).

### 1.3. Mitophagy

**Figure 5:**
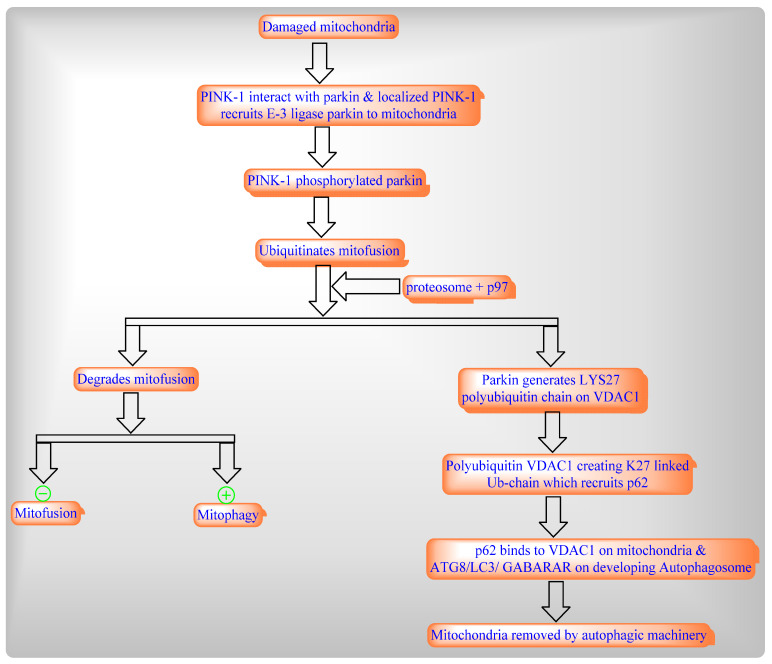
Damaged-Induced Mitophagy involves two major proteins: [1] ubiquitin kinase PINK1 (which flags the damaged mitochondria); and [2] parkin, E_3_ ligase (as a signal amplifier).

**Figure 6:**
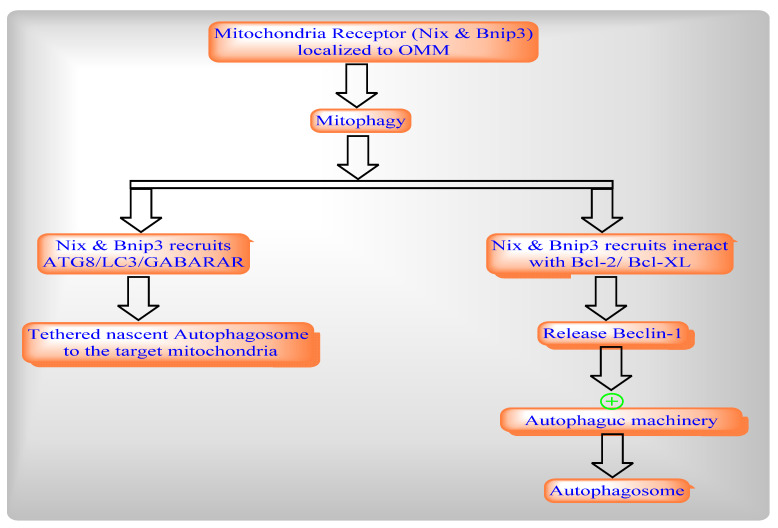
Development-Induced Mitophagy.

Mitophagy is selective autophagy process in mitochondria which includes the rapid removal of dysfunctional or damaged mitochondria through two different pathways such as damage-induced mitophagy (Figure 5) and development-induced mitophagy (Figure 6).

In healthy mitochondria, PINK1 contains a mitochondrial target sequence (MTS), which translocates to mitochondria and is imported to the IMM by translocase of the outer mitochondrial membrane (OMM) and inner mitochondrial membrane (TIM). Following this, PINK1 is degraded by downstream proteolytic events.

In damaged mitochondria, loose-membrane potential accommodates TIM and TOM activity resulting in the stabilization of PINK1 on the OMM of damaged mitochondria [78,79], and engages parkin ubiquitin ligase, which is activated by phosphorylation and deubiquitination. Therefore, PINK1 and parkin selectively tagged damaged mitochondria with a ubiquitin chain engulfed by phagophore to forma mitophagosome. As a result, this mitophagosome fused with the lysosome and damaged mitochondria that were delivered to the lysosome. Activated PINK1 requires the recruitment of optineurin (OPTN) and NDP52, whereas parkin does not require autophagy recruitment. PINK1 generates phospho-ubiquitin, which serves as a unique signature for the recruitment of the mitophagy receptor protein and parkin to build the ubiquitin chain for signal amplification [80].

Parkin/PINK1 also promotes TB1 activation and enhances ubiquitin chain building [81,82].

#### The Pathological Role of Mitophagy Development

Mitophagy destructs paternal mitochondria in fertilized oocytes. During fertilization in mammals, paternal sperm-born mitochondria (ubiquitin+) enter the ooplasm and are degraded by the ubiquitin–proteasome system [83].

In mammals, Nix selectively removes paternal mitochondria. Many ubiquitinated membranous organelles (MOs) degrad paternal mitochondria with the help of autophagy [84]. During aging, the autophagy gene and related proteins decrease in humans and mice [85,86,87]. A condition such as caloric restriction delays the aging-related degeneration process by activating autophagy. Mitophagy decreases ROS production and removes dysfunctional mitochondria [88]. Autophagy acts as a tumor suppressor in human cancers such as breast, prostate, and ovarian cancer, where the autophagy gene Beclin1 is deleted [89]. Thus, the loss of autophagy enhances tumorigenesis. Autophagy is positively regulated by tumor-suppressor genes such as Lkt, AMPK, and Pten [90,91,92,93]. In limited nutrients or oxygen in tumor tissues, autophagy acts as a buffer to metabolic stress.

Mutation in PINK1 and parkin causes Parkinson’s disease. Alzheimer’s disease occurs due to mitochondrial dysfunction and defective cytochrome [94] as β-amyloid fragments target mitochondria, whereas in HD (Huntington’s Disease) occurs due to dysregulated PGC1-α, which is an important transcription factor for mitochondrial biogenesis [95]. Moreover, aging causes mitochondrial dysfunction and weaknesses in skeletal muscle functions due to the deterioration of mitochondrial signaling. Improvement in mitochondrial function enhances immunity which prevents the spreading of viruses. Targeting mitochondrial dynamics and processes may be beneficial for treatments against COVID-19 and other viruses [96,97].

### 1.4. The Relation between Mitochondrial Fission and Fusion, Apoptosis and Mitophagy

**Figure 7:**
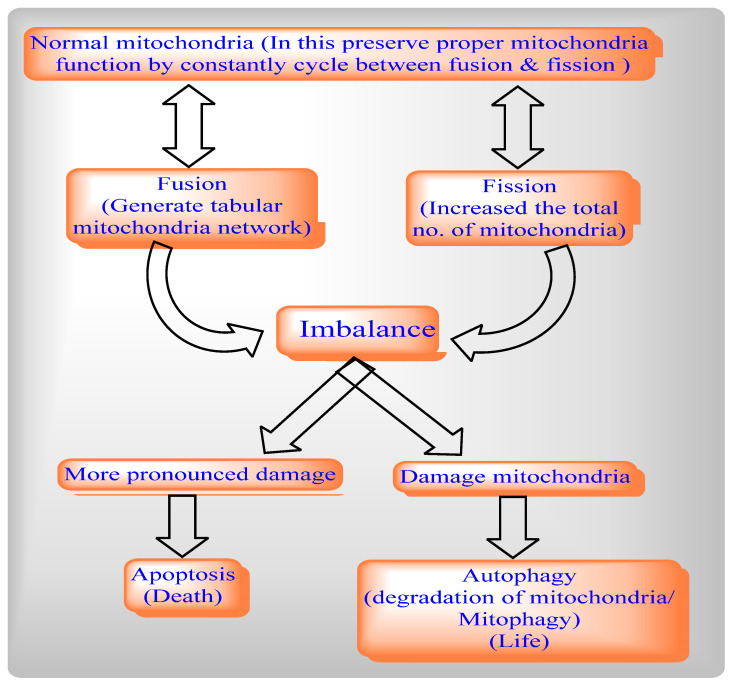
Relationships between mitochondrial fission and fusion, apoptosis and mitophagy.

In normal conditions, mitochondrial preserve their overall shape and function via maintaining a balance between mitochondrial fusion and fission. Mitochondrial fusion fuses healthy mitochondria with functional tubular mitochondrial network by isolating dysfunctional mitochondria, whereas mitochondrial fission increases the total number of mitochondria. During viral infection, the balance between mitochondrial fusion and fission is disturbed which leads to mitophagy, whereas in cases of more pronounced damage it leads to mitochondrial-dependent apoptotic cell death (Figure 7). Thus, viruses modulate various functions like autophagy and mitophagy to propagate their replication during viral infection (Table 2). We will continue to develop effective therapeutic strategies for virotherapy by understanding role of autophagy from the perspective of individual viruses.

## 2. Viruses and Their Effects on Mitochondrial Metabolites

In the host cell, viruses use building blocks such as lipids and amino acids for their virion progeny production, whereas energy causes processes such as viral assembly and release [113,114,115]. Moreover, mitochondria have evolved antiviral counter measures. Viruses mainly influence two different mitochondrial metabolic pathways such as the β-oxidation of fatty acids and the Tricarboxylic acid cycle or Krebs Cycle (Figure 7). Mitochondria are clustered around the replication sites of several viruses and decrease the supply routes for energy and metabolites, resulting in increased viral progeny viruses. In a viral infection, viruses generate cellular stress, which causes mitochondrial redistribution.

Slow-replicating viruses target the mitochondria by maintaining cellular energy homeostasis to ensure efficient replication and an extended lifecycle, also avoiding programmed cell death. In contrast, fast-replicating viruses easily cope with cellular metabolic dysfunction.

### 2.1. Regulation of Ca^2+^ Homeostasis by Viruses in Host Cells

Involved in various cellular process, Ca^2+^ acts as secondary messenger. Among different mechanisms, Ca^2+^ can enter through voltage-dependent anion channels [VDAC), also known as mitochondrial porins in outer membrane, into the mitochondrial intermembrane space [116,117]. This channel regulates Ca^2+^ entry and metabolites based on mitochondrial membrane potential (MMP). Ions such as Na^+^, H^+^, and Ca^2+^ exchange across the mitochondrial membrane resulting in decreased MMP, which depends upon the electron transport chain (ETC). The permeability transition pore (PTP) regulates Ca^2+^ efflux via a “flickering” mechanism. In Ca^2+^ overload, the PTP are opened for a longer duration which causes the destruction of mitochondrial functions. In the inner-mitochondrial membrane, oxidative stress, Ca^2+^ overload, and ATP depletion induce the formation of a non-specific permeability transition pore (PTP), which is also responsible for damage to the MMP. Moreover, viruses regulate MMP in the host cells. The MMP value varies from species to species and organ to organ, based on mitochondrial function, protein composition, and the amount of oxidative phosphorylation activity required in that organ of the body [118].

At the early stage of virus infection, viruses prevent apoptosis from resulting in the prevention of the host immune response and promote cell replication. On the opposite side, at a later stage of virus infection, viruses induce apoptosis and release the progeny virions for dissemination to the surrounding cells.

### 2.2. Role of Viruses in Modulating Mitochondrial Antiviral Immunity

Viruses attack cells to generate interferon via activating a variety of signal transduction pathways. Pathogen-associated receptors (PRRs) such as the toll-like receptor (TLRs), nucleotide oligomerization domain (NOD) like receptor [NLRs), and retinoic acid-inducible gene (RIG-I) like receptor (RLRs), recognize the pathogen-associated molecular atoms (PAMPs) of viruses which are present inside the cell. PRRs directly activate immune cells [119].

Mitochondria are associated with RLRs such as the melanoma differentiation-associated gene 5 (Mda-5) and retinoic acid-inducible gene I [RIG-I), which recognize the dsRNA. RIG-I has two terminuses. The N-terminus contains caspase activation and recruitment domains (CARDs) and includes proteins such as mitochondrial antiviral signaling (MAVS), IFN-β promoter stimulator 1 (IPS-1), virus-induced signaling adaptor (VISA), or the CARD adaptor-inducing IFN-β (CARDIF) protein. On the other hand, the C-terminus includes RNA helicase activity [120] which binds to unmodified RNA produced by a viral polymerase in an ATPase-dependent manner, resulting in the exposure of its CARD domain and activating a downstream effector which leads to the formation of enhanceosome-triggering [121] NF-kB production.

Mitochondrial Antiviral Signaling (MAVS) contains a proline-rich region on the N terminal CARD and the hydrophobic transmembrane (TM) on the C-terminal, which targets the protein in the mitochondrial outer membrane [122]. Thus, it plays an essential role in antiviral defense in the cells. The overexpression of MAVS activates NF-kB and IRF-3, which produce type 1 interferon responses. By interacting MAVS with VDAC [123] preventing apoptosis and the opening of MPTP, the virus cleaves the MAVS from the mitochondrial outer membrane and reduces interferon response [124,125].

For example:

1. HCV cleaves MAVS in amino acids (508) and paralyzes the host defense against HCV;
2. The flaviviridae GB virus B, NH3/4A protein cleaves MAVS and prevents any interferon product [126]. MAVS is associated with RLRs which produce type 1 interferon [IFNs] and pro-inflammatory cytokines [127] that act against the pathogen interferon regulatory factor (IRF) and produce type 1 IFN in the cytoplasm [128,129]. Peroxisomal MAVS are involved in the induction of IFN-stimulated genes like encoding viperin [130].

### 2.3. AMPK Governs Autophagy and Mitochondrial Homeostatsis

**Figure 8:**
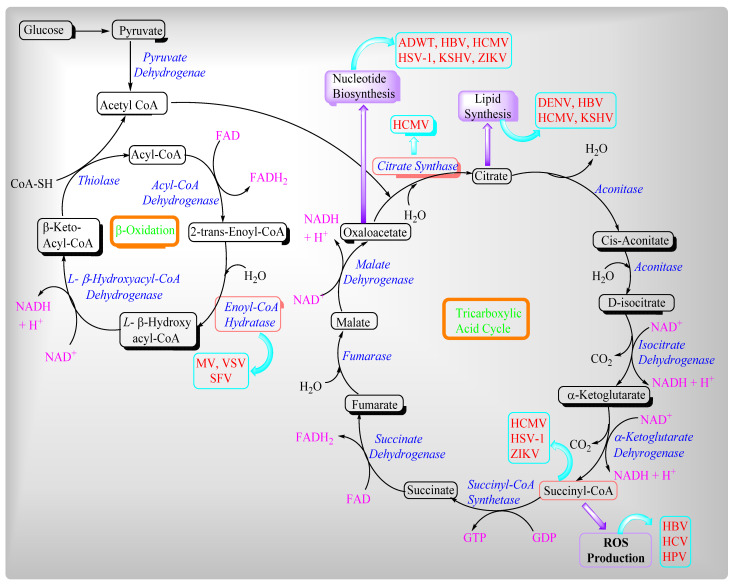
Viruses’ influence on beta-oxidation and the TCA cycle.

The AMP-activated protein kinase (AMPK) complex consists of different subunits including a catalytic α-subunit and two regulatory subunits, β and γ. The AMPK complex senses low cellular ATP levels to increase growth control nodes and the phosphorylation of specific enzymes which produce ATP or lower ATP consumption. AMPK plays an important role in multiple biosynthetic pathways under low cellular energy levels via direct and indirect targeting of the functions of different protein targets of AMPK (Figure 8).

### 2.4. Role of SRV2 in Mitochondrial Dynamics

Ras val-2 (SRV2) is a pro-fission protein that promotes interaction between Drp1 and mitochondria [131], then oligomerizes Drp1 around mitochondria to form a ring and cut the mitochondria into several fragments. Thus, it has a vital role in mitochondrial shape and fission [132]. The protein SRV2 also increases the expression of F-actin (as stress fiber) and it provides an adhesive force which helps Drp1 to complete mitochondrial contraction [133,134] which facilates mediated mitochondrial fission [135]. Macro phase stimulating 1 (Mst 1) is a key factor in the Hippo signaling pathway. The loss of Mst 1 maintained mitochondrial homeostasis [136] by the attenuation of renal ischemia-reperfusion injury as well as in cardiomyocytes, improving mitochondrial performance by autophagy and enhanced cardiomyocyte viability. Additionally, Mst 1 has a role in SRV2-related mitochondrial fission.

#### 2.4.1. SRV2 in Various Functions of Mitochondria

Mitochondrial fission is promoted by the LPS-mediated upregulation of SRV2 [137,138]. Loss of SRV2 attenuates mitochondrial fission, protects cardiomyocytes against LPS-induced stress, and improves cell survival and sustained cardiomyocyte function [139].

SRV2 overexpression promotes mitochondrial fission and leads to cardiomyocyte death and mitochondrial damage [140]. Thus, the loss of SRV2 exerts an antioxidative effect in cardiomyocytes by inhibiting mitochondrial fission.

With regard to mitochondrial ETC activity, the knockdown of SRV2, LPS, and FCCP have similar effects and decrease ETC transcription. The inhibition of mitochondrial fission prevents the LPS-induced dysregulation of cardiomyocyte energy metabolism [141,142,143].

#### 2.4.2. Relationship between Mitochondria, Oxidative Stress, and Inflammation in COVID-19

**Figure 9:**
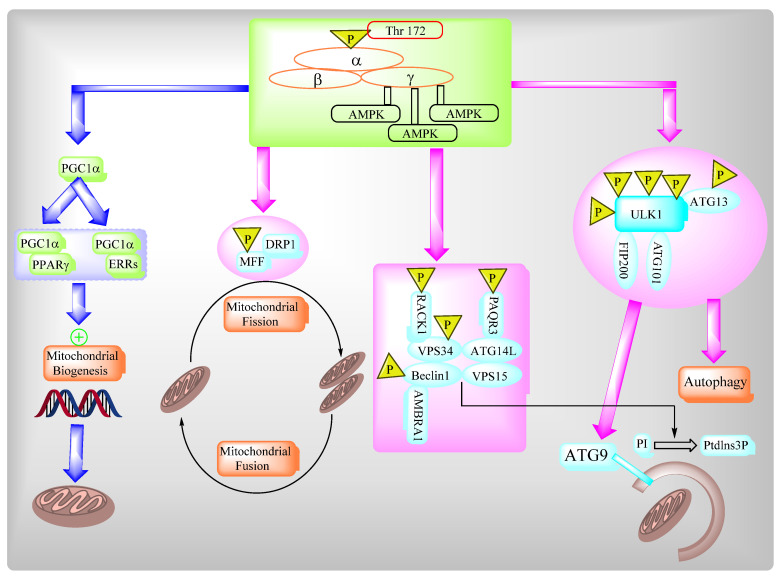
AMPK regulates a variety of metabolic processes.

**Figure 10:**
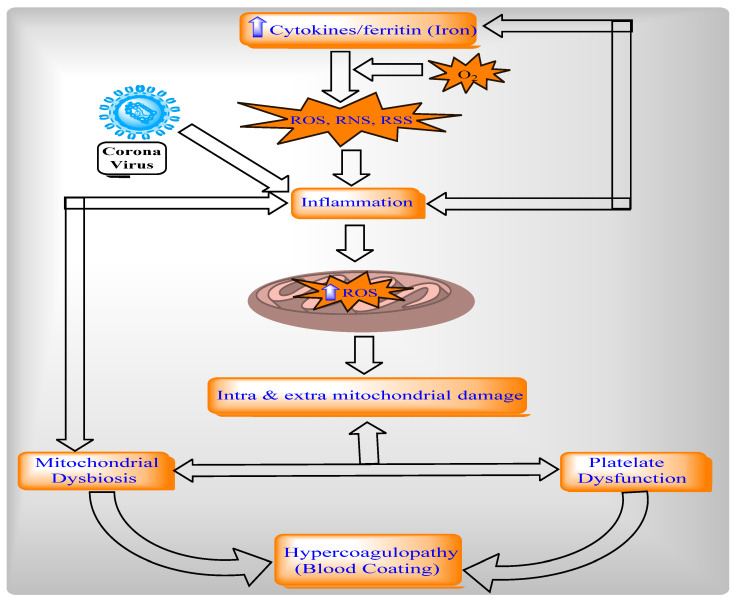
Mitochondria dysfunction in the pathogenesis of COVID-19.

The protein ROS increases via inflammatory cytokines, such as TNF-alpha in mitochondria, and directly stimulates a generation of pro-inflammatory cytokines [144]. The ROS in mitochondria is modulated by IL-6 and IL-10. Mitochondrial metabolism is altered through intracellular cascades, which are triggered by inflammatory mediators and immune sentinels. The serum of patients with COVID-19 contains cytokines like TNF- alpha and IL-6, which obstruct mitochondrial oxidative phosphorylation, ATP production, and produce ROS in the cell [145,146]. These ROS-altered mitochondrial dynamics permeabilize the mitochondrial membrane and ultimately cause cell death. Additionally, ROS production and mitochondrial content (such as mtDNA) are released into the cytosol and the extracellular environment [147,148]. After this, ROS activates NLRP3 inflammasomes and produces pro-inflammatory cytokines such as IL-1beta and induces the production of IL-6 via inflammasome-independent transcriptional regulation [145,146,149,150]. Thus, ROS contributes to mitochondrial dysfunction (Figure 9 and Figure 10). Cytokines can indicate COVID-19 disease severity. Patients with COVID-19 have a large number of pro-inflammatory cytokines (CXCL-8, IL-6, CCL3, CCL4, and IL-12) due to human alveolar epithelial cells with dysfunctional mitochondria [151]. Thus, these cells impair repair responses and reduce responsiveness to corticosteroid (Figure 10).

### 2.5. Different Pathways to Reposition Common Approved Drugs against COVID-19

The World Health Organization reported that most repositioned drugs modulators, under clinical investigation against COVID-19, act through different pathways such as UPR, autophagy, the NLRP3 inflammasome, and mitochondrial permeability transition pores [MPTP] (Table 3).

## 3. Expert Opinion

Mitochondria are membrane-bound cell organelles which produce energy in the form of adenosine triphosphate (ATP) as well as regulating various intracellular functions like metabolism, bioenergetics, cell death, innate immune signaling, and cellular homeostasis. Mitochondria are self-governed by mitochondrial dynamics and mitochondria-selective autophagy or mitophagy. During infection, viruses altered mitochondrial dynamics in order to modulate mitochondria-mediated antiviral immune responses via the alteration of mitochondrial events such as autophagy, mitophagy, and cellular metabolism to facilitate their proliferation.

The pro-fission protein of SRV2 activates mitochondrial fission via the loss of MMP, the ROS-overloading suppression antioxidant system, the depletion of cellular ATP, the release of the apoptotic factor, the activation of the caspase family, and NLRP3 inflammasomes. The protein SRV2 also promotes mitochondria-associated cardiomyocyte apoptosis to cause cardiomyocyte death and mitochondrial damage. The World Health Organization reported that most repositioned drugs modulators, under clinical investigation against COVID-19, act through different pathways such as UPR, autophagy, the NLRP3 inflammasome, and mitochondrial permeability transition pores (MPTP) to inhibit SARS-COV2 propagation. Analysis of the functional significance of mitochondrial dynamics and viral pathogenesis will open up new possibilities for the therapeutic design of approaches to combat viral infections and associated diseases. 

## Figures and Tables

**Table 1:** Effect of the autophagy inducer and inhibitor on the replication of coronavirus in cell cultures.

Viruses Species	Drugs		Mechanism of Action at Various Step of Autophagy
	Autophagy Inducers	Autophagy Inhibitor	
SARS-CoV-2 [54]	Ivermectin		Ivertine inhibited SARS-CoV-2 by inhibiting the AKT phosphorylation [55].
SARS-CoV-2 [56]		Nitazoxanide/Alinia	These drugs also inhibit SARS-CoV-2 by the blocking of late- stage lysosome acidification [57].
[56,58]		Chloroquine	Chloroquine increses the PH of lysosome and prevents the formation of autolysosome [59], thus it inhibits SARS-CoV-2 virus.
SARS-CoV [60]	Valinomycin		Valinomycin stimulates mitophagy by loss of MMP, Ref. [61] causing it to inhibit the replication of SARS-CoV virus.
	Aescim		Aescim inhibits SARS-CoV virus by activating the signaling pathway of ROS-MAPK/p38 [62].
[63,64]		Chloroquine	Chloroquine acts similary to SARS-CoV-2 and inhibits the SARS virus [59].
MERS-CoV [65]	Venetoclax		Venetoclax inhibits MERS-CoV by releasing the BECN1 from BCL2L1 or by interacting with BCL2L1/Bcl-X_L_ [66].
	Everolimus/Afinitor		These drugs inhibit MTOR causing it to inhibit the MERS-CoV Virus [67].
[68]	Rapamycin/Sirolumus		These drugs act in the same way as Everolimus and Afinitor and inhibit virus repliucatin [69].
		wortmannin	Wortmannin inhibits MERS-CoV by inhibiting the PtdIns3K & PI3K_S_ [70].
		UO126	It inhibits the MAPK/ERK pathway and therefore inhibits the MERS-CoV virus [71].
MHV [72]	Rapamycin/Sirolumus		It also acts in a similar way to the MERS-CoV virus [69].
[73]		3-MA	It acts by inhibiting the class III PtdIns3K and therefore inhibits the MHV virus [74].

**Table 2:** Viruses and their effects on mitochondrial dynamics.

S.No.	Author & Year	Virus	Work & Object
1	Horner and Gale, 2013 [98]	Hepatitis C virus (HCV)	HCV cleaves the MAVS protein and suppresses the host’s antiviral response.
2	Datan et al., 2016 [99], Liang et al., 2016 [100]	Dengue and Zika virus	With the help of autophagy, the Dengue and Zika viruses improve their replication and the induction of autophagy by pharmacological agents (e.g., rapamycin) increasing viral dissemination.
3	Joubert et al., 2012 [101]	Chikungunya virus	Autophagy limits virus-induced cell death and in vivo mortality in Chikungunya virus.
4	Datan et al., 2016 [99]; Lee et al., 2008 [102]; McLean et al., 2011 [103]	Dengue virus	Autophagy inhibits apoptosis to enhance virus replication in the Dengue virus.
5	Zhu et al., 2016 [104]	Transmissible gastroenteritis virus (TGEV)	TGEV-induced complete mitophagy by stimulating DJ1-1 protein deglycase which increases cell survival and infection by eliminating virus-induced ROS.
6	Meng et al., 2014 [105]	Newcastle disease virus (NDV)	Delayed administration of 3 methyl adenine (3-MA) induced more efficient oncolysis in NSCLCs.
7	Barbier et al., 2017 [106]	Dengue virus	In the Dengue virus, mitochondrial fission is blocked because the Dengue virus’ NS4B or NS3 protein promotes mitochondrial fusion by downregulating Drp1.
8	Yu et al., 2015 [107]	Dengue virus	In the case of the Dengue virus, mitochondrial fusion is suppressed by NS2B3 protease which cleaves MFNs.
9	Zamarin et al., 2005 [108]	Influenza A virus	PB1-F2 have an essential role in the pathogenicity of the viral infection of influenza virus A, via modulation of the host’s mitochondrial dynamics.
10	Kim et al., 2013b [109]	Hepatitis C virus (HCV)	HCV stimulates the expression of parkin, PINK1 and induced mitophagy by impairing oxidative phosphorylation. The resulting HCV infection affects mitochondrial dynamics.
11	Gou et al., 2017 [110]	Classical swine fever virus (CSFV)	CSFV expresses MFN2 and stimulates parkin and PINK1 expression, resulting in enhanced mitochondrial fission and mitophagy.
12	Ding et al., 2017 [111]	Human parainfluenza virus type 3 (HPIV3)	In HPIV3 infection, a viral protein regulates mitophagy independently of parkin/PINK1.
13	Xia et al., 2014b [112]	Measles virus	During the measles viral infection, virus-induced antiviral immune response is enhanced by the knockdown of autophagy-related genes (eg, ATG7, BECN1, SQSTM1, and RAB7).

**Table 3:** List of drugs which targeted SARS- CoV related pathways.

Therapeutic Category	Mechanism of Action						
	Autophagy			UPR stress		MPTP	NLRP3 Inflammasome
	Activator	Modulator	Inhibitor	Suppressor	Modulator	Modulator	Inhibitor
Immunosuppressant	Rapamycin, Tacrolimus, Everolimus [152]					Cyclosporin A [153,154]	
Anticancer	Rapamycin, Tersirolimus, Everolimus [152], Gefitinib [155], Temozolomide [155]		Bortezomib, Celecoxib [155]	Sunitinib [156]			Thalidomide [157]
Antidiabetic	Metformin [152]			Pioglitazone [156], Exenatide, Vildagliptin [158], Berberine [159]	Liraglutide [159]		Glyburide [157,160]
Dietary supplement	Trehalose, Resveratro l [152]			Curcumin [156]	Quercetin [161]		
Antipsychotic	Lithium [152], Fluspirilene, Trifluperazine, Pimozide [162], Bromperidol, Chlorpromazine [163,164], Sertindole, Olanzapine, Fluphenazine, Methotrimeprazine [165], Prochlorperazine [164]	Clozapine [165]				Haloperidol [166,167,168], Etifoxine [169,170]	
Antiepileptic	Carbamazepine, Sodium valproate [152]						
Antihypertensive	Verapamil, Nimodipine, Nitrendipine [152], Nicardipine, Amidarone [162], Rilmenidine, Clonidine [171], Minoxidil [163]			Isoproterenol [156], Valsartan, Lowsartan, Olmesartan, Telmisartan [158], Guanabenz [172], Bisoprolol, Propranolol, Metoprolol [159]		Ifenprodil [166,167,168], Diazoxide, Nicorandil, Tadalafil, Perhaxiline, Carvedilol [153,154]	
Antidiarrheal	Loperamide [162]						
Ca^+^ regulator	Calcifediol [171]						
Anti-infective	Nitazoxanide [171]						
Antidepressant	Nortriptyline [171]		Clomipramine [163]	Trazodone [173]			
AnticholesteremiC agent	Simvastatin [170]				Atorvastatin [159]		
Antiemetic	Chlorpromazine [163,164], Prochlorperazine [164]					Haloperidol [166,167,168]	Thalidomide [157]
Minercorticoid replacement agent	Fludraocortisone [163,164]						
Antitussive	Noscapine [163,164]					Carbetapentane, Dextromethorphan [166,167,168]	
Anti-allergic	Clemastine [163]						
Chelating agent	Defeiprone [174]						
Antihelmintic	Niclosamide [175]		Quimacrine [176,177]				
Skeletal muscle relaxant		Baclofen [178]					
Gastrointestinal			Pantoprazole [155]				
Macrolide antibiotic			Azithromycin [163]				
Ocular drug			Verteporfin [163]				
Antiprotozoal drug			Quimacrine [176,177], Chloroquine, Hydroxychloroquine [171]				
Urea cycle disorder agent				Thenylbutyrate [155,156]			
Hypolipidemic agent				Pravastatin [156], Fenofibrate [158]			
Anti-Alzheimer’s						Donepeziol [166,167,168]	
Anti-Parkinsonian						Pramipexole [179]	
Neuroprotective agent; anti-ALS drug						Edaravone [153,180]	
Anti-arthritic							Anakinra [157]
Anti-inflammatory agent			Celecoxib [155]				Anakinra [157], Tranilast [157,181]
Anti-insomia agent					Melatonin [182]		
